# Risk of Subsequent Preeclampsia by Maternal Country of Birth: A Norwegian Population-Based Study

**DOI:** 10.3390/ijerph20054109

**Published:** 2023-02-25

**Authors:** Karolina S. Mæland, Nils-Halvdan Morken, Erica Schytt, Vigdis Aasheim, Roy M. Nilsen

**Affiliations:** 1 Faculty of Health and Social Sciences, Western Norway University of Applied Sciences, 5063 Bergen, Norway; 2 Department of Clinical Science, University of Bergen, 5007 Bergen, Norway; 3 Center for Fertility and Health, Norwegian Institute of Public Health, 0213 Oslo, Norway; 4 Department of Obstetrics and Gynecology, Haukeland, University Hospital Bergen, 5021 Bergen, Norway; 5 Center for Clinical Research Dalarna, Uppsala University, 791 82 Falun, Sweden

**Keywords:** preeclampsia, immigration, pregnancy, country of birth, recurrence, barrier

## Abstract

In this nationwide population-based study, we investigated the associations of preeclampsia in the first pregnancy with the risk of preeclampsia in the second pregnancy, by maternal country of birth using data from the Medical Birth Registry of Norway and Statistics Norway (1990–2016). The study population included 101,066 immigrant and 544,071 non-immigrant women. Maternal country of birth was categorized according to the seven super-regions of the Global Burden of Disease study (GBD). The associations between preeclampsia in the first pregnancy with preeclampsia in the second pregnancy were estimated using log-binomial regression models, using no preeclampsia in the first pregnancy as the reference. The associations were reported as adjusted risk ratios (RR) with 95% confidence intervals (CI), adjusted for chronic hypertension, year of first childbirth, and maternal age at first birth. Compared to those without preeclampsia in the first pregnancy, women with preeclampsia in the first pregnancy were associated with a considerably increased risk of preeclampsia in the second pregnancy in both immigrant (*n* = 250; 13.4% vs. 1.0%; adjusted RR 12.9 [95% CI: 11.2, 14.9]) and non-immigrant women (*n* = 2876; 14.6% vs. 1.5%; adjusted RR 9.5 [95% CI: 9.1, 10.0]). Immigrant women from Latin America and the Caribbean appeared to have the highest adjusted RR, followed by immigrant women from North Africa and the Middle East. A likelihood ratio test showed that the variation in adjusted RR across all immigrant and non-immigrant groups was statistically significant (*p* = 0.006). Our results suggest that the association between preeclampsia in the first pregnancy and preeclampsia in the second pregnancy might be increased in some groups of immigrant women compared with non-immigrant women in Norway.

## 1. Introduction

Preeclampsia is a pregnancy complication affecting 3 to 5% of women globally [1,2]. It is a leading cause of perinatal morbidity and mortality [2] as well as a risk factor for adverse long-term maternal health consequences including cerebrovascular and cardiovascular diseases [3,4]. Although the exact cause of preeclampsia is unknown, its risk strongly increases with higher maternal age, body mass index, interpregnancy weight change, gestational diabetes, and chronic hypertension [5,6]. Recent research has further highlighted an increased risk of preeclampsia in women with COVID-19 infection in early pregnancy [7]. Additionally, a genetic predisposition appears to increase the risk; women experiencing preeclampsia in a first pregnancy have a significantly increased risk of preeclampsia in a second pregnancy compared with those who do not develop the condition in the first pregnancy [8,9].

Previous studies of preeclampsia suggest that immigrant women overall have a lower risk of preeclampsia than women in the host population in the receiving countries [10,11,12,13]. This has been largely explained by the healthy migrant effect, in that women migrating from one country have better health at arrival than the general population in the receiving country [10,14]. However, more recent studies using maternal country of birth as the exposure show a more nuanced picture, with a higher risk of preeclampsia in refugees and women from low-income countries [13,15]. Thus, to better understand the variation in preeclampsia risk across immigrant groups in receiving countries, alternative hypotheses should be investigated.

In Norway, antenatal care services are offered free of charge and the use of interpreters is statutory [16,17]. However, previous studies suggest that subgroups of immigrant women giving birth in receiving countries may not receive intelligible information and recommendations given during pregnancy and childbirth [18,19]. They also report a low usage of interpreters in maternity care and difficulties navigating the healthcare system to gain information and receive appropriate care during pregnancy [18,19]. Due to such structural barriers to access healthcare, immigrant women may receive poorer quality of care during pregnancy compared with non-immigrants. It is therefore conceivable that some subgroups of immigrants may also be susceptible to complications and health problems during pregnancy.

As part of the postpartum follow-up program in Norway, all women with preeclampsia in a pregnancy should be informed of the high recurrence risk of preeclampsia in a subsequent pregnancy [20]. They should further be advised to avoid general risk factors for preeclampsia such as high interpregnancy weight gain [5]. However, if structural barriers reduce access to healthcare, this information may not be given or correctly understood, reducing the possibility to prevent preeclampsia in a subsequent pregnancy. If this information is not communicated in a tailored and intelligible manner in maternity care for immigrant women, we might expect a higher risk of recurrent preeclampsia in some immigrant groups compared with non-immigrant women.

To test this hypothesis and to identify the subgroups of immigrant women susceptible for preeclampsia, we examined the association of preeclampsia in a first pregnancy with the risk of preeclampsia in the second pregnancy across seven maternal regions of birth as defined by the Global Burden of Disease study (GBD).

## 2. Materials and Methods

### 2.1. Study Design

This population-based registry study used individual-linked data from the Medical Birth Registry of Norway (MBRN) and Statistics Norway. The linkage of data and identification of all pregnancies to the same woman was enabled through the national identity number assigned to all Norwegian residents. The MBRN comprises mandatory, standardized notification of all live- and stillbirths from 16 weeks of gestation (12 weeks since 2002) in Norway since 1967 [21]. The data include information on maternal health before and during pregnancy, and information on maternal and infant health during pregnancy, labor, and birth [21]. Statistics Norway collects, processes, and distributes official statistics in Norway [22]. Data comprise sociodemographic and migration-related factors about all individuals who are or have been a resident in Norway since 1990 [23].

### 2.2. Study Sample

**Figure 1:**
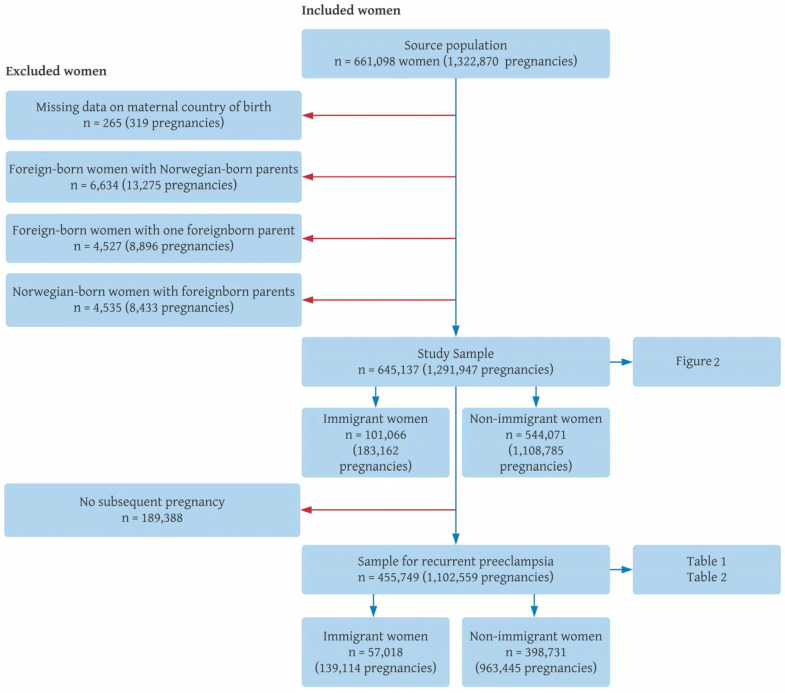
Derivation of the study sample, Norway, 1990–2016.

We analyzed all women with first and subsequent births from 1990 to 2016 (*n* = 661,098 women with 1,322,870 pregnancies). In particular, women giving birth before 1990 or having their first child outside of Norway during the study period (i.e., women registered as multiparous at the first registered pregnancy in the MBRN) were not included in the initial source population. Furthermore, we focused our analyses only on women categorized as immigrant women (foreign-born with two foreign-born parents) and non-immigrant women (Norwegian-born with at least one Norwegian-born parent). Foreign-born women with one foreign-born parent and those born in Norway to two foreign-born parents (second generation immigrants) were not analyzed as these represented smaller heterogeneous groups. After performing these exclusions, our study sample contained 645,137 women with 1,291,947 pregnancies (Figure 1).

### 2.3. Preeclampsia

Preeclampsia was based on coding according to the International Statistical Classification of Disease and Related Health Problems, 8th (1990–98) and 10th revisions (1999 onwards). This coding corresponds with the criteria given by the Norwegian Society of Gynecology and Obstetrics, i.e., an increase in blood pressure (≥140/90 mmHg) combined with proteinuria (≥300 mg in a 24 h urine collection) after 20 weeks of gestation [20,24]. The diagnosis was recorded in the MBRN by open text (1990–1998) or by checkbox (from 1999 onwards). Validation studies covering two periods (1967 to 2005 and 1999 to 2010) [25,26] indicate that the registration of preeclampsia correlates well with medical records.

### 2.4. Region of Birth

Maternal country of birth was obtained from Statistics Norway. Due to the small numbers of preeclampsia in both the first and second pregnancies in the study population, we categorized maternal country of birth (immigrant women only) according to the seven super-regions defined by the GBD study [27,28] as follows: (i) Central Europe, Eastern Europe, and Central Asia; (ii) high income; (iii) Latin America and the Caribbean; (iv) North Africa and the Middle East; (v) South Asia; (vi) Southeast Asia, East Asia, and Oceania; and (vii) Sub-Saharan Africa. The high income regions contained women from the following countries: Southern Latin America, Western Europe, North America, Australasia, and high income Asia Pacific [28].

### 2.5. Other Variables

The MBRN also provided information on maternal age at birth (in years), year of childbirth, parity, and interpregnancy interval (in months). The interpregnancy interval was calculated as the time between the birth of a first child to an estimated conception of a second child (time of birth minus gestational age) to the same woman [29]. Length of residence (immigrants only) was calculated as the difference between year of childbirth of the first child (data from the MBRN) and year of official residence permit in Norway for the mother (data from Statistics Norway).

### 2.6. Statistical Analyses

All analyses were performed in Stata IC version 16 (Stata Statistical Software, College Station, TX, USA), using women as the study unit of analysis. Women with multi-fetal pregnancies were counted only once.

The analyses were organized in two parts (see Figure 1). First, we described absolute preeclampsia risk in first pregnancy and absolute recurrence risk in subsequent pregnancies up to the fourth pregnancy in the source population (*n* = 1,291,947 pregnancies). We additionally calculated the numbers for each subsequent pregnancy in these analyses. All calculations were performed separately for immigrants and non-immigrants overall and the results were visualized in a tree diagram using the approach by Hernández-Díaz et al. [8].

In the second part and the main analysis, we compared the risk of preeclampsia in the second pregnancy given preeclampsia status in the first pregnancy for women with at least two pregnancies and for each of the seven maternal GBD regions of birth (*n* = 1,102,559 pregnancies). Investigations of preeclampsia risk beyond the second pregnancy were not performed due to limited preeclampsia numbers for several immigrant groups of higher parities. The associations were estimated using log-binomial regression models and reported as crude and adjusted risk ratios (RRs) with 95% confidence intervals (CIs), adjusted for chronic hypertension, year of first childbirth, and maternal age at first birth.

Finally, to investigate if the RR of preeclampsia in a second pregnancy after preeclampsia in the first pregnancy differed across the seven GBD regions, a likelihood ratio test was performed by comparing the log-likelihood for a model with and without an interaction term (preeclampsia in first pregnancy × GBD super-regions). A significant interaction term would indicate different effect estimates across groups.

In the sensitivity analyses, we excluded women with multi-fetal pregnancies and HELLP syndrome (hemolysis, elevated liver enzymes, and low platelet count). We also performed additional adjustments for education, interpregnancy interval, and length of residence (immigrants only) to account for other possible background differences between groups. We further adjusted for maternal body mass index for the years available (2008–2016) for immigrant and non-immigrant women overall. The results remained essentially the same.

### 2.7. Ethics and Public Involvement

This is an observational study approved by the Southeast Regional Committees for Medical and Health Research Ethics in Norway; reference number: 2014/1278/REK Southeast Norway. Data were used under license for this study.

This study used standardized surveillance data. Patients were not involved in the development of the research question, outcome measures, design, or conduct of the study.

## 3. Results

The overall risk of preeclampsia in the study was 3% (5% in the first pregnancy and 2% in later pregnancies). The risk of preeclampsia in the first pregnancy for immigrants and non-immigrants was 2.9% (*n* = 2965) and 4.8% (*n* = 26,125), respectively.

Table 1 shows the relevant background characteristics in the sample of women with at least one subsequent pregnancy. Among immigrants, women from high income regions represented the largest group (*n* = 13,508 women) while the smallest group comprised women from Latin America and the Caribbean (*n* = 1445 women).

**Figure 2:**
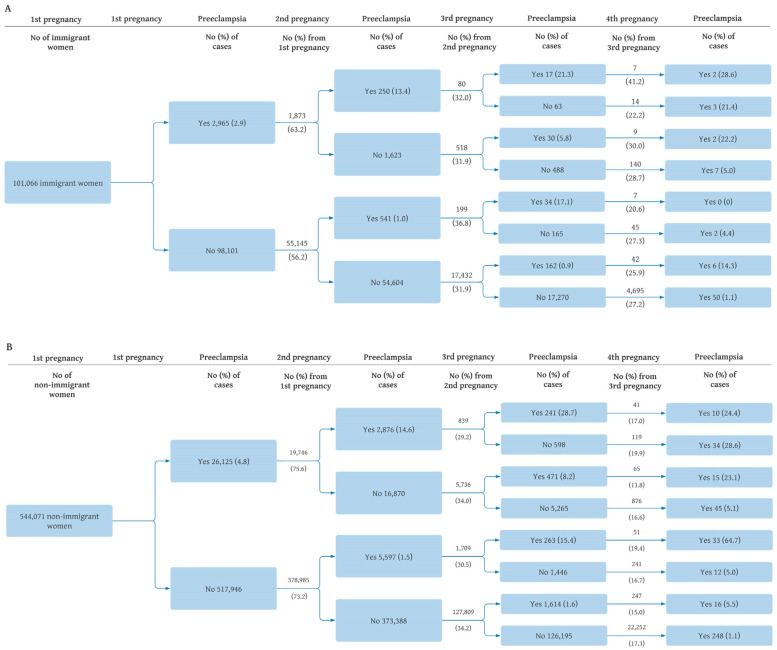
Risk of preeclampsia, up to four subsequent pregnancies in immigrant (**A**) and non-immigrant (**B**) women, Norway, 1990–2016.

Figure 2 presents the risks of preeclampsia for up to four subsequent pregnancies in immigrant (Figure 2A) and non-immigrant (Figure 2B) women. Among those with preeclampsia in the first pregnancy, the risk of preeclampsia in the second pregnancy was 13.4% (*n* = 250) for immigrants and 14.6% (*n* = 2876) for non-immigrants. For women with a third pregnancy, the risk of preeclampsia in all three subsequent pregnancies was 21.3% for immigrants and 28.7% for non-immigrants (Figure 2).

The mean maternal age at first birth ranged from 24.9 [SD 3.9] to 29.9 [SD 4.4] years in immigrant women from South Asia and the high-income regions, respectively. Among women with two or more pregnancies, mean parity ranged from 2.2 [SD 0.5] in immigrant women from Latin America and the Caribbean to 2.8 [SD 1.1] in immigrant women from Sub-Saharan Africa. The mean interpregnancy interval between the first and second pregnancy ranged from 24 months [SD 22.6] in Sub-Saharan immigrants to 35 months [SD 29.4] in women from Latin America and the Caribbean.

Table 2 shows the crude and adjusted RR for preeclampsia in the second pregnancy for women with preeclampsia in the first pregnancy compared with women without preeclampsia in the first pregnancy. Immigrant women from Latin America and the Caribbean had the highest RR of preeclampsia in the second pregnancy (adjusted RR 17.4 [95% CI 8.1–37.4]), followed by immigrant women from North Africa and the Middle East (adjusted RR 14.9 [95% CI 10.5–21.3]). The lowest RR of preeclampsia in the second pregnancy was found in non-immigrant women (adjusted RR 9.5 [95% CI 9.1–10.0]). The difference in RR across regions of birth was statistically significant by the likelihood ratio test in both crude (*p* = 0.004) and adjusted (*p* = 0.006) regression models.

In immigrant women, those with preeclampsia in the first pregnancy were more likely to proceed with a second pregnancy compared with those who did not develop preeclampsia in the first pregnancy (Figure 2; 63% and 56%, respectively), but no apparent group difference was seen for later pregnancies. For non-immigrant women, the likelihood of a second pregnancy was almost similar for those with and without preeclampsia in the first pregnancy (Figure 2; 76% and 73%, respectively), but fewer women with previous preeclampsia had a third pregnancy (29% and 34%).

When excluding women with multi-fetal pregnancies (*n* = 26,086) and women with HELLP syndrome (*n* = 683), the results in Table 2 remained essentially the same. Furthermore, additional adjustment for education, interpregnancy interval, and length of residence (immigrants only) did not affect the results notably.

## 4. Discussion

In this study, we found that all women who experienced preeclampsia in the first pregnancy had a substantially increased risk of preeclampsia in the second pregnancy compared with women without preeclampsia in the first pregnancy, irrespective of the country of birth. We further showed that this association was stronger for immigrant women overall as well as for certain subgroups of immigrant women compared with non-immigrant women.

Our finding of a stronger association with preeclampsia in immigrant women compared with non-immigrant women may support our predefined hypothesis of the current study. The importance of follow-up and tailored information is crucial to reduce the subsequent risk of pathology in pregnancy [30]. All women developing preeclampsia in Norway should be carefully informed about the recurrence risk before entering a subsequent pregnancy [20]. They should also be advised not to gain interpregnancy weight as this increases the risk of recurrent preeclampsia [5]. Moreover, women with a history of preeclampsia should be advised to control their blood pressure early in a subsequent pregnancy [20]. This information is essential to increase the awareness of possible lifestyle adjustments and for the early detection of preeclampsia in subsequent pregnancies. However, due to possible structural communication barriers between immigrant women and the healthcare system [18,19], we hypothesized that immigrant women with preeclampsia in a first pregnancy to a lesser extent than non-immigrants receive or acquire sufficient preventive information on recurrent preeclampsia in a subsequent pregnancy. If our hypothesis is true, we therefore would expect a higher risk of subsequent preeclampsia in some immigrant groups compared with others.

To our knowledge, this is the first study to compare the RR for preeclampsia in a subsequent pregnancy between immigrant and non-immigrant women. Being the first study, the discussion of our results in comparison to previous studies is therefore challenging. However, in light of our hypothesis, it may be more interesting to compare our results with results from countries that immigrant women in Norway frequently migrate from. If the RR of subsequent preeclampsia in immigrant women in a receiving country is higher compared with the RR of data from a woman’s country of birth, our hypothesis of poorer communication in receiving countries may be supported. For example, in a hospital-based study in Tanzania, the RR of preeclampsia in a second pregnancy was reported to be 9-fold for women with a history of preeclampsia compared with those without a history [31]. In our study, we found that immigrant women from the Sub-Saharan African region overall had an almost 11-fold increased risk of preeclampsia in a second pregnancy. A higher RR in immigrant women compared with non-immigrant women may support our hypothesis of poorer communication between immigrant women and healthcare providers.

Although our results could support the communication barrier hypothesis, findings should be discussed in light of the large RR and their CIs. When comparing the RR across GBD regions, the RR varied from 10 to 18. However, the CI for these effect estimates largely overlapped the RR of non-immigrant women (see Table 2), except for immigrant women from North Africa and the Middle East (RR 15) as well as immigrant women from high income countries (RR 14). Further, when analyzing immigrants overall, we found that the RR for subsequent preeclampsia for immigrants and non-immigrants was 13 and 10, respectively. Despite the higher RR for preeclampsia in immigrants compared with that of non-immigrants, the RRs are large and the difference in RR between the groups is relatively small. We therefore should be careful to firmly conclude that immigrant women with preeclampsia in a first pregnancy are susceptible to a higher risk of preeclampsia in a second pregnancy compared with non-immigrant women.

Because our study did not directly measure the hypothesized communication barriers, we cannot be entirely certain that the difference in the RR between immigrants and non-immigrants is truly caused by poorer communication between immigrants and healthcare providers. There might be other potential mechanisms for the observed differences, including a genetic susceptibility for increased preeclampsia in some immigrant groups that we were not able to control for in our analyses. Further, the complexity of migration should not be underestimated [32,33] and the stressors related to the process of migration, i.e., unsafe migration routes, could have had an impact on our results. However, despite not accounting for these mechanisms, we would expect that the RR for some immigrant groups was lower than that found for non-immigrants. Instead, our results showed a consistently higher RR for all studied GBD groups, which may strengthen the hypothesis of communication barriers in immigrant women compared with Norwegian-born women.

Consistent with previous studies [11,13,15], we found that the overall risk of preeclampsia (the proportion of preeclampsia across all parities) was lower in immigrant than in non-immigrant women (3% vs. 5%). The lower overall risk of preeclampsia in immigrant women compared with non-immigrants has mainly been explained by the healthy immigrant effect [12], in that women moving to another country are healthier than the general population in the receiving country [34]. In this study, focusing on the preeclampsia risk in the second pregnancy given preeclampsia status in the first pregnancy, it appears that immigrants do not have a lower RR for preeclampsia in a second pregnancy. A plausible explanation for the diverging results of overall and subsequent risk of preeclampsia may relate to the genetic aspect of preeclampsia. Those who develop preeclampsia in a first pregnancy are at a genetically high risk of developing the condition in a subsequent pregnancy for both immigrant and non-immigrant women, irrespective of the healthy migrant effect.

Awareness of the risk of subsequent preeclampsia and preventive measures to reduce this risk in the second pregnancy is crucial for women with preeclampsia in the first pregnancy. Tailored information on the importance of follow-up during pregnancy to obtain the best compliance in maternity care is hence crucial for immigrant women. The main strengths of this study include the national population-based design, the standardized collection of data, and the large sample size. The large sample size and the long timespan of the study enabled a detailed analysis on the risk and subsequent risk for both immigrants and non-immigrants over time. By using the unique personal identification number, all pregnancies to the same woman were identified and enabled an accurate calculation of risk and subsequent risk up to a fourth pregnancy. Previous validation studies of preeclampsia diagnosis in the MBRN [25,26] have reported that the diagnosis correlates well with medical records, adding further strength to our study.

This study has some limitations. Because of the low number of recurrent preeclampsia cases in most countries, we grouped our study sample into broad GBD regions. This may have led to an underestimation or overestimation of the risk of preeclampsia for immigrant women from a specific country, which may further reduce generalizability to specific immigrant groups.

## 5. Conclusions

In this national population-based study of women with two or more pregnancies, both immigrant and non-immigrant women with preeclampsia in a first pregnancy had a substantially increased risk of preeclampsia in a second pregnancy compared with those without preeclampsia in a first pregnancy. The variation between GBD regions overall was not that strong; however, immigrant women from some GBD regions appeared to have a higher risk of preeclampsia in a second pregnancy than non-immigrants. Close follow-up for all women with a history of preeclampsia is important for early detection and possible treatment of the condition in a subsequent pregnancy.

## Figures and Tables

**Table 1:** Background characteristics by maternal region of birth for women with two or more pregnancies, Norway, 1990–2016.

Maternal Characteristic	Non-Immigrants	Central Europe, Eastern Europe, Central Asia	High Income Countries	Latin America, Caribbean	North Africa, Middle East	South Asia	Southeast Asia, East Asia, Oceania	Sub-Saharan Africa
**No. of women** (%)	398,731 (87.5)	12,151 (2.7)	13,508 (3.0)	1445 (0.3)	9340 (2.1)	4641 (1.0)	9721 (2.1)	6212 (1.4)
**Maternal age** ^a^ (mean ± SD)	26.2 ± 4.5	26.6 ± 4.4	29.9 ± 4.4	28.3 ± 4.9	25.2 ± 4.6	24.9 ± 3.9	26.9 ± 4.4	25.8 ± 4.6
**Year of childbirth** ^a^ (mean ± SD)	2001 ± 7.0	2007 ± 5.8	2003 ± 7.0	2005 ± 6.0	2004 ± 6.3	2002 ± 6.9	2002 ± 6.8	2006 ± 6.3
**Parity** ^b^ (mean ± SD)	2.4 ± 0.7	2.3 ± 0.6	2.3 ± 0.6	2.2 ± 0.5	2.6 ± 0.8	2.8 ± 1.0	2.4 ± 0.6	2.8 ± 1.1
**Interpregnancy interval** ^a,c^								
months (mean ± SD)	33.3 ± 26.6	31.0 ± 24.4	27.2 ± 20.8	35.1 ± 29.4	31.2 ± 26.3	27.7 ± 23.4	33.1 ± 26.1	24.3 ± 22.6
**Length of residence** ^a,d^								
years (mean ± SD)	-	4.1 ± 5.1	5.5 ± 5.6	3.8 ± 4.6	4.2 ± 5.7	6.1 ± 7.8	5.0 ± 6.2	3.6 ± 4.5
**Maternal education** ^a,e^ (%)								
No education	0.0	0.3	0.2	0.9	3.0	2.0	1.3	8.1
Primary education	18.9	18.8	9.9	22.0	43.7	39.9	34.0	50.1
Secondary education	37.9	29.5	25.3	24.4	27.8	27.3	29.7	24.6
University/college	43.2	51.5	64.6	52.7	25.6	30.8	35.0	17.3
Missing ^b^	0.2	22.9	15.3	25.3	36.5	31.5	24.9	33.5

SD: standard deviation. ^a^ Reported for 1st pregnancy. ^b^ Mean for women with at least two pregnancies. ^c^ Missing interpregnancy interval (*n* = 1058). ^d^ Missing length of residence (*n* = 645). ^e^ Missing educational level (*n* = 15,230).

**Table 2:** Risk ratios (RRs) with 95% confidence intervals (CIs) for preeclampsia in second pregnancy by maternal region of birth, Norway, 1990–2016.

Maternal Region of Birth	No. of Women ^a^	Preeclampsia in Second	Crude RR (95% CI)	Adjusted RR (95% CI) ^b^
	No.	No. (%)		
**Total Sample**				
No preeclampsia in first	434,130	6138 (1.4)	1.00 (Reference)	1.00 (Reference)
Preeclampsia in first	21,619	3126 (14.5)	10.2 (9.82, 10.7)	9.8 (9.4, 10.2)
**Immigrant**				
No preeclampsia in first	55,145	541 (1.0)	1.00 (Reference)	1.00 (Reference)
Preeclampsia in first	1873	250 (13.4)	13.6 (11.8, 15.7)	12.9 (11.2, 14.9)
**Non-Immigrant**				
No preeclampsia in first	378,985	5597 (1.5)	1.00 (Reference)	1.00 (Reference)
Preeclampsia in first	19,746	2876 (14.6)	9.86 (9.45, 10.3)	9.5 (9.1, 10.0)
**Central Europe, Eastern Europe, and Central Asia**				
No preeclampsia in first	11,831	82 (0.7)	1.00 (Reference)	1.00 (Reference)
Preeclampsia in first	320	34 (10.6)	15.3 (10.4, 22.5)	14.1 (9.7, 20.7)
**High Income Countries**				
No preeclampsia in first	12, 993	124 (1.0)	1.00 (Reference)	1.00 (Reference)
Preeclampsia in first	515	73 (14.2)	14.9 (11.3, 19.6)	14.5 (11.0, 19.1)
**Latin America and Caribbean**				
No preeclampsia in first	1396	14 (1.0)	1.00 (Reference)	1.00 (Reference)
Preeclampsia in first	49	9 (18.4)	18.3 (8.33, 40.3)	17.4 (8.1, 37.4)
**North Africa and Middle East**				
No preeclampsia in first	9092	87 (1.0)	1.00 (Reference)	1.00 (Reference)
Preeclampsia in first	248	38 (15.3)	16.0 (11.2, 22.9)	14.9 (10.5, 21.3)
**South Asia**				
No preeclampsia in first	4493	61 (1.4)	1.00 (Reference)	1.00 (Reference)
Preeclampsia in first	148	23 (15.5)	11.5 (7.29, 18.0)	10.6 (6.8, 16.6)
**Southeast Asia, East Asia, and Oceania**				
No preeclampsia in first	9447	103 (1.1)	1.00 (Reference)	1.00 (Reference)
Preeclampsia in first	274	32 (11.7)	10.7 (7.34, 15.6)	10.4 (7.2, 15.2)
**Sub-Saharan Africa**				
No preeclampsia in first	5893	70 (1.2)	1.00 (Reference)	1.00 (Reference)
Preeclampsia in first	319	41 (12.9)	10.8 (7.48, 15.6)	10.4 (7.2, 15.0)

^a^ Women with at least one subsequent birth included (*n* = 455,749). ^b^ Adjusted for chronic hypertension, year of first childbirth, and maternal age at first birth.

## Data Availability

Data are available from the corresponding author upon request and with permission from the Medical Birth Registry of Norway and Statistics Norway.
